# Future career intentions of recent GP graduates in Ireland: a trend analysis study

**DOI:** 10.3399/bjgpopen18X101409

**Published:** 2018-04-21

**Authors:** Ivana Pericin, Gerard Mansfield, James Larkin, Claire Collins

**Affiliations:** 1 Research Assistant, Research Department, Irish College of General Practitioners, Dublin, Ireland; 2 GP, Sheehan Medical Practice, Dublin, Ireland; 3 Research Assistant, Research Department, Irish College of General Practitioners, Dublin, Ireland; 4 Director of Research, Research Department, Irish College of General Practitioners, Dublin, Ireland

**Keywords:** general practice, GP graduates, career intentions, emigration, part-time employment

## Abstract

**Background:**

A lack of manpower and negative health statistics have increased the workload for Irish GPs. Consequently, recent GP graduates are considering emigration or part-time employment.

**Aim:**

To report on trends of the current status and future work intentions of recent GP graduates in Ireland.

**Design & setting:**

Quantitative study based on online surveys in the Irish setting.

**Method:**

A ‘career intentions’ survey was emailed to all recent GP graduates in Ireland, in 2014, 2015, and 2017. The data presented includes GPs who graduated in the previous 4 years at each survey time point. The average response rate across the three surveys was 38.2%.

**Results:**

The number of graduates who had already emigrated increased over the survey years, accounted for 16.9% in 2014, 17.4% in 2015, and 19.2% in 2017 survey. The majority of doctors who emigrated did so in the first 2 years after graduation (74.7%). ‘Quality of life’ became the most frequent reason for emigration over the survey years, accounting for 32.0% in the 2017 survey. In the 2014 survey, 47.3% of graduates stated that they intend to work part-time in 5 years; this rose to 51.2% in 2015, reaching 60.0% in 2017. Female participants were more than twice as likely to plan to work part time in 5 years compared to their male colleagues, across all three surveys.

**Conclusion:**

The first and second years after graduation were the most critical for emigration. Interventions in this period may reduce the 'brain drain' of Irish GPs. Part-time working is becoming more attractive and should be considered in future workforce planning.

## How this fits in

Brain drain is affecting medicine and general practice internationally. Previous research shows that, as a result of work–life imbalance, future GPs consider emigration and part-time employment as an option.

This study outlines trends in GP emigration and future working intentions, which have substantial impact on the future planning of general practice in Ireland. This study will be of interest to other counties which are experiencing similar trends.

## Introduction

The model where GPs hold a central role in the provision of primary health care is commonly applied in Ireland and internationally.^1–8^ The EU countries with a strong primary care structure, including Denmark, Finland, and the Netherlands, often emphasise the role of the GP as a gatekeeper, and as the provider of a wide range of health services.^3,5,6^ Since the population in Ireland is aging, with increasing numbers of patients with chronic disease,^2,9^ the demand for GPs is anticipated to grow. However, general practice continues to face numerous challenges where, due to an inadequate level of state support, workforce shortages and brain drain are taking place.

The contemporary state general medical services (GMS) contract is a contract-for-service introduced in 1989.^2,10^ Its main focus is on the provision of acute care in general practice, but it neglects care for chronic conditions.^2,10,11^ It is the principal means through which the state provides resourcing for general practice services, and is available to eligible members of the population. Clinical limitations arise from a lack of resources to manage chronic diseases, including limited access to diagnostics by GPs.^12^ The GMS contract is, financially, almost entirely capitation based, with no resourcing made available to deliver care to modern standards. This antiquated reimbursement scheme produces high levels of dissatisfaction among Irish GPs, who have called for an urgent revision.^13^ In addition, the Financial Emergency Measures in the Public Interest Act (FEMPI), introduced in 2009, meant a vast resource reduction for practices.^14^ As a result, many GPs have considered emigration as an option, indicating Australia and Canada as preferred destinations.^15^ These countries have greater numbers of GPs per capita,^16^ fewer working hours per week, and higher annual incomes for GPs.^17^ The workforce crisis in general practice was also noted by other organisations, including the Health Service Executive (HSE) and the Irish Medical Organisation (IMO), which highlighted that Ireland faces shortages in GPs and that investment in general practice is vital.^1,18^

In order to foresee a potential crisis and inform policy decisions, it is important to gain an insight into the career plans of the future GP workforce in Ireland. This study does so by presenting survey trends among recent Irish GP training graduates since 2014.

## Method

A ‘career intentions’ survey was undertaken by the Irish College of General Practitioners (ICGP), targeting recent GP graduates in Ireland, in 2014, 2015, and 2017. In Ireland, a GP graduate represents a doctor who, after gaining an undergraduate medical degree, has undergone general practice specialist training, which consists of 2 years of a hospital training post followed by 2 years in supervised general practice.^19^ The ICGP provides education and training in general practice and is the membership body for GPs in Ireland, and therefore holds the database of all GP trainees as well as its GP members, which constitute >85% of all qualified GPs in Ireland.^20^ Therefore, the ICGP was able to send an online survey via email using Survey Monkey to graduates from the respective years. A follow-up reminder was issued 1 week later to all non-responders. The surveys were emailed to 1647 GP graduates in total, accounting for 457 graduates in 2014, 608 graduates in 2015, and 582 graduates in 2017. While the initial surveyed population varied in terms of inclusion, the data presented here includes GPs who graduated in the previous 4 years at each survey time point in order to allow for comparison (the survey from 2014 included graduation years from 2010–2013; the 2015 survey included 2011–2014; and the 2017 survey included 2013–2016). It is important to stress that, since a range of the graduation years overlap in each of the survey years, there is a possibility that the opinions from the same graduates could have appeared multiple times through the analysis.

The aim of the survey was to investigate employment status as well as the emigration plans of responders. The first part of the surveys included questions focused on demographics data (sex, age, and year of graduation), followed by the current employment status and location, and future intentions in terms of emigration and employment. The employment status was categorised through the number of sessions per week, where part-time employment was defined as <8 sessions per week, and full-time employment as ≥8 sessions per week. The questions that investigated graduates' perspectives and preferences about general practice were additionally incorporated in the 2015 and 2017 surveys, and were not part of the 2014 questionnaire (further information available from the authors on request). Responses for these questions were collected on a 5-point scale including ‘strongly agree’, ‘agree’, ‘neutral’, ‘disagree’, and ‘strongly disagree’. To enable the use of χ^2^ analysis by avoiding small cell counts, and to simplify the tabulation of data, this scale was reduced to a 3-point scale (with ‘strongly agree’ and ‘agree’ combined). The data was analysed using SPSS Statistics (version 23).

## Results

### Survey distribution and response rates

Overall, 629 graduates responded, representing an aggregated response rate of 38.2%. The data included is for GPs who graduated in the previous 4 years at each survey time point, and therefore consists of 244 responders in 2014, 164 responders in 2015, and 144 responders in 2017.

### Emigration

#### GP graduates residing abroad at the time of the surveys

The survey results showed that the emigration rates of the GP graduates have slightly increased over the years. According to the survey in 2014, 16.9% (*n *= 41) of GP graduates were abroad at the time of survey, in comparison with 19.2% (*n* = 25) who participated in the survey from 2017 (see Table 1).

**Table 1.** Demographics, including sex and year of graduation, of GP graduates who had already emigrated at the time of the survey

	Year of survey		
	2014, *n* (%)	2015, *n* (%)	2017, *n* (%)
Already emigrated	41 (16.9)	28 (17.4)	25 (19.2)
**Sex**			
Male	18 (23.4)	12 (20.3)	7 (17.1)
Female	23 (14.4)	15 (15.2)	18 (20.2)
**Year of graduation**			
2010	11 (20.4)		
2011	14 (19.4)	8 (20.0)	
2012	7 (14.9)	4 (14.8)	
2013	9 (13.0)	12 (24.0)	8 (21.6)
2014		4 (9.1)	6 (14.0)
2015			10 (24.4)
2016			1 (11.1)

**Figure 1.**
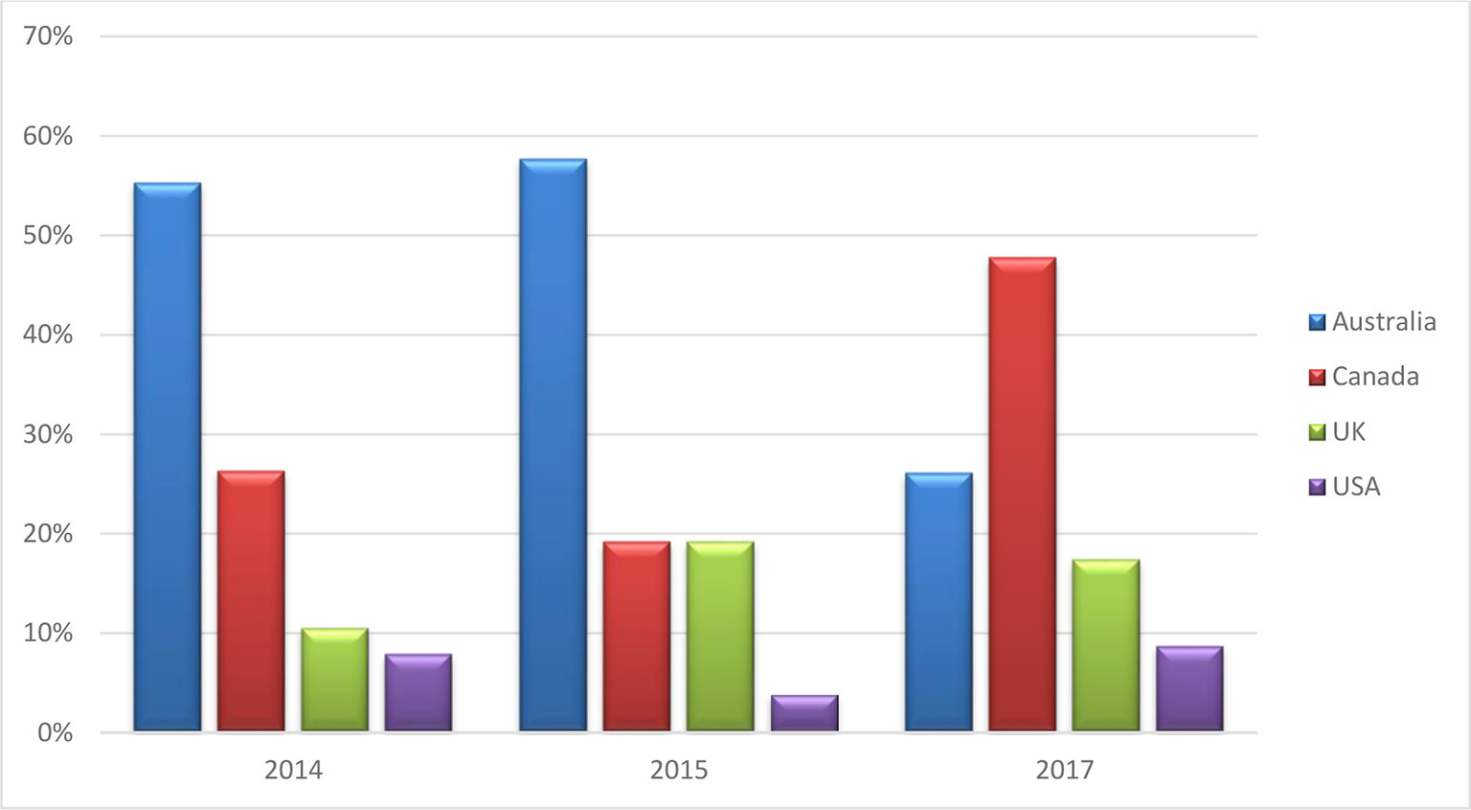
Current location of GP graduates who had already emigrated at the time of the survey.

Australia and Canada were found to be the most preferable destinations for those who had already emigrated over all 3 survey years. However, a slight change in preferable destinations was noted. While the majority of those who emigrated were located in Australia in the 2014 (55.3%, *n* = 21) and 2015 (57.7%, *n* = 15) surveys, by the 2017 survey, the number of GP graduates in Australia declined sharply, accounting for 26.1% (*n* = 6). Australia was replaced by Canada, which became the most preferable destination according to the 2017 survey, accounting for 47.8%, (*n* = 11) of GP graduates located abroad (Figure 1). Although there was a numerical difference in the preference of destination, it was not possible to do a significance test due to the small expected cell counts.

**Figure 2.**
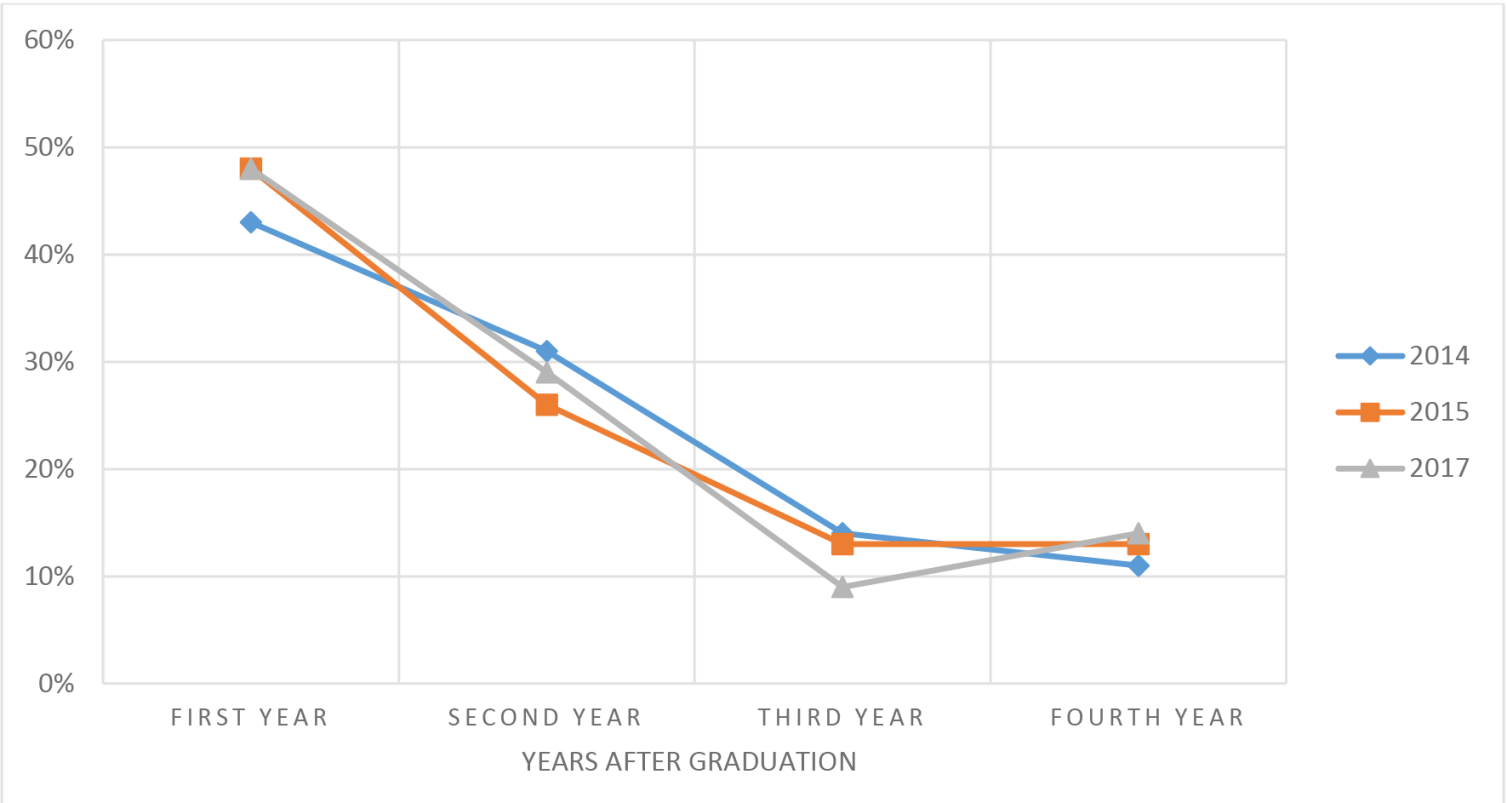
Year of emigration after graduation among GP graduates who had already emigrated at the time of the survey.

Aggregated data across all three surveys revealed that 45.6% (*n* = 36) of GP graduates who were abroad at the time of surveys emigrated in the first year after graduation, and 74.7% (*n* = 59) emigrated within the first 2 years (Figure 2). The number of graduates who emigrated in the third year after graduation across all three survey years accounted for 12.7% (*n* = 10), and 12.7% (*n* = 10) for the fourth year.

To examine potential reasons for emigration, responders who had already emigrated were given a choice to select one of the following: ‘financial prospects’, ‘quality of life’, ‘professional satisfaction’, ‘partner’s working requirement’, ‘lack of structure around partnership/succession’, ‘concerns regarding viability of the general practice’, and ‘other’ (see Table 2). ‘Quality of life’, ‘partner’s working requirement’, and ‘financial prospects’ were equally preferable in the survey from 2014, with 22.5% (*n* = 9) of responders choosing each reason. However, by the 2017 survey ‘quality of life’ (32.0%, *n* = 8) was most often selected as the main reason for emigration. ‘Concerns regarding viability of general practice’ was the least cited, and showed a sharp decline from 17.5% (*n* = 7) in the 2014 survey, to 4% (n = *1*) in the 2017 survey (see Table 2). The proportion who plan to return to Ireland ranged from 17.1–23.1%, with an aggregated average of 19.1% (*n* = 18) of graduates who are abroad planning to return to Ireland.

**Table 2.** Reasons for emigration of GP graduates who have already emigrated.

	Year of survey		
	2014, *n* (%)	2015, *n* (%)	2017, *n* (%)
Concern regarding viability of general practice	7 (17.5)	3 (12.5)	1 (4.0)
Financial prospects	9 (22.5)	7 (29.2)	5 (20.0)
Lack of structure around partnership/succession arrangements	4 (10.0)	1 (4.2)	0 (0.0)
Partner's working requirement	9 (22.5)	7 (29.2)	5 (20.0)
Professional satisfaction	2 (5.0)	0 (0.0)	4 (16.0)
Quality of life	9 (22.5)	6 (25.0)	8 (32.0)
Other	1 (2.5)	0 (0.0)	2 (8.0)
Total,* n*	40	24	25

#### GP graduates who were in Ireland at the time of surveys

The participants were asked ‘Do you plan to emigrate in the near future?’ and the option to select one of the following was given: ‘No’, ‘Yes, possibly’, ‘Yes, definitely’, and ‘Undecided’. The majority of GP graduates who were still residing in Ireland at the time of the surveys planned to stay and continue their work engagements in Ireland (53.2%, *n* = 232). However, in the region of one-fifth stated that they definitely or possibly plan to emigrate in the future; this figure shows a reduction over time from 26.8% (*n* = 53) in 2014 to 19.2% (*n* = 20) in 2017 (Table 3). The majority of GP graduates who were undecided about their emigration plans, cited ‘family reasons’ as the main reason for staying in Ireland. This was stated by 63.3% (*n* = 31) of undecided responders in 2014, 52.0% (*n* = 14) in 2015, and 55.0% (*n* = 11) in 2017. Future emigration plans, as well as destination preference, were not significantly related to any of the measured demographics; that is, sex, age, relationship status, having children, or year of graduation (significance tests available from author on request).

**Table 3.** Emigration plans of recent GP graduates who currently reside in Ireland.

	Year of survey			
	2014, *n* (%)	2015, *n* (%)	2017, *n* (%)	All years, %
Emigration plans				
Yes, definitely	19 (9.6)	13 (9.7)	9 (8.7)	9.4
Yes, possibly	34 (17.2)	20 (14.9)	9 (8.7)	14.4
Undecided	53 (26.8)	27 (20.1)	20 (19.2)	23.0
No	92 (46.5)	74 (55.2)	66 (63.5)	53.2
Total, *n*	198	134	104	436

### Perspectives and plans towards employment

The surveys from 2015 and 2017 investigated in more depth the perspectives of recent GP graduates towards their role and their views on the responsibilities of a GP in the practice. The GP graduates were presented with the statements including: ‘I find the traditional responsibilities of a practice principal/partner attractive. These include accountability for financial, property, and employment coordination of the whole practice’ and ‘As a GP, I would like to focus on the clinical aspects of the job exclusively’. Options such as ‘strongly agree’, ‘agree’, ‘neutral’, ‘disagree’, and ‘strongly disagree’ were given. These questions were not offered in the 2014 survey.

The surveys displayed that a high number of recent graduates are reluctant to take up the traditional responsibilities of the practice. The percentage of participants who disagreed or strongly disagreed that being accountable for finances, property, and employment coordination of the whole practice is attractive was 44.5% (*n *= 68) in 2015 and 47.0% (*n *= 55) in 2017. When combining responses from 2015 and 2017, it was found that 58.0% of those who definitely or possibly plan to emigrate disagreed or strongly disagreed with this statement, compared to 40.9% of those who don’t plan on emigrating. However, a χ^2^ test revealed that this was not a statistically significant difference (χ^2^ [2 degrees of freedom {df}] = 5.160, *P = *0.076).

The majority of graduates indicated that they agree or strongly agree that they would like to focus on the clinical aspects of the job exclusively, accounting for 57.9% (*n* = 88) in the 2015 survey and 51.3% (*n* = 60) in the 2017 survey. Furthermore, in relation to emigration plans, it was found that 61.2% of those who definitely or possibly plan to emigrate agreed or strongly agreed with this statement, compared to 50.4% of those who don’t plan on emigrating, although, a χ^2^ test revealed that this was not a statistically significant difference (χ^2^ [2 df] *= *2.303*, P = *0.316).

**Figure 3.**
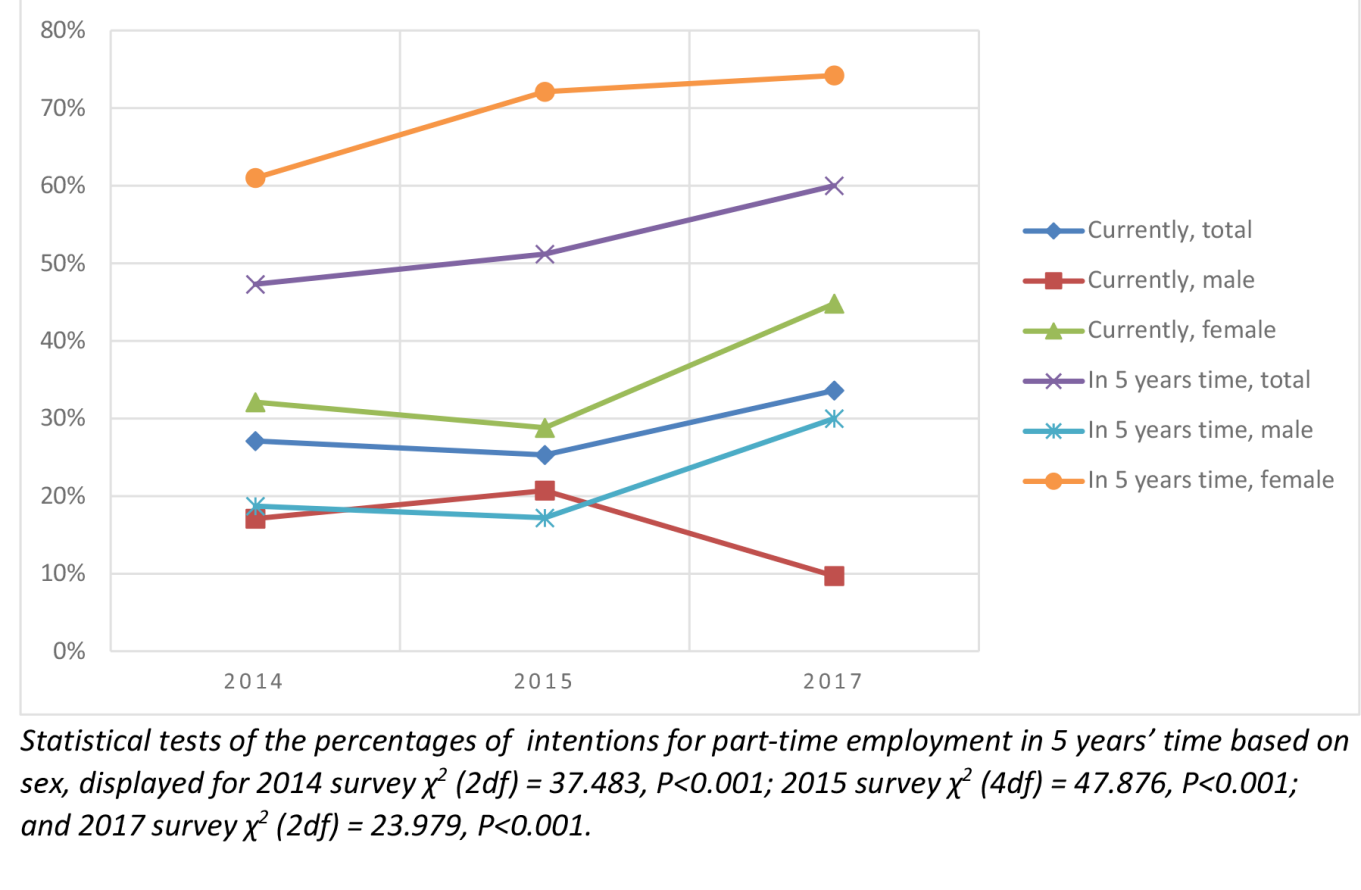
Part-time employment of recent GP graduates based on sex.

In terms of employment hours, the majority of responders worked full time after graduation: 65.7% (*n* = 155) in 2014; 68.4% (*n* = 108) in 2015; and 62.5% (*n* = 80) in 2017. However, when examining their predictions for the future, both male and female graduates across all survey years envisage having fewer working hours. In the 2014 survey, 47.3% (*n* = 113) of graduates intended to work part-time hours in 5 years’ time. The anticipation of part-time employment rose over the years, reaching 60.0% (*n* = 75) in the 2017 survey (Figure 3). A χ^2^ test revealed a statistically significant relationship between sex and part-time employment (*P*<0.001), where female responders were more than twice as likely to plan to work part time in 5 years (61.0% [*n* = 97] in 2014, 72.1% [*n* = 70] in 2015, to 74.2% [*n* = 63] in 2017 survey) in comparison with their male colleagues (18.7% [*n *= 14] in 2014, 17.2% [*n* = 10] in 2015, to 30.0% [*n* = 12] in 2017 survey) (Figure 3). The analysis of the 2015 and 2017 survey revealed that 73.6% of GP graduates who intended to work part time in 5 years’ time disagreed or strongly disagreed that being accountable for finances, property, and employment coordination of the whole practice is attractive, in comparison to 61.2% of those who intended to be full-time employed. The statistical tests revealed that there was a significant relationship between intention to work part-time in the future and expanded GP role (χ [1 df] = 4.640, *P *= 0.031). The GPs who intended to work part time in the future were more likely to have a negative attitude towards being accountable for finances, property, and employment coordination of the whole practice.

## Discussion

### Summary

In recent years, general practice in Ireland has experienced workforce changes and challenges. In order to plan for the future and contribute to policy decisions, an insight into the overall intentions of newly qualified GPs is essential. The number of graduates who already emigrated at the time of the survey remained relatively constant over the years, 16.9% in 2014 with a slight increase in 2017 of <3%. Almost three-quarters of doctors who emigrated (74.7%) did so in the first 2 years after graduation, and therefore these initial years were found to be the most critical in terms of emigration. By 2017, ‘quality of life’ (32.0%) was the most dominant reason which influenced graduates’ decision to leave the country. Australia and Canada were found to be the commonest destinations of those who emigrated, with Canada becoming more popular in recent years, possibly due to tightening Australian immigration laws.^21^ The analysis of the surveys also indicated that recent graduates do not agree with the current changes in general practice, in terms of both the clinical and non-clinical aspects. A high number of responders (>50% in both the 2015 and 2017 survey) are more interested in being purely a clinician, and do not desire senior management roles and the responsibility for the practice infrastructure. Due to the FEMPI cuts, practices had no control over increased care demand being placed on reduced infrastructure,^15,22^ which changed the workforce environment for new and established GPs alike.

Trends over time showed an increase in intentions to work part time in the future, especially among female doctors. In the 2017 survey, in comparison with previous survey years, there was an increased number of graduates (60%) who anticipated working <8 sessions per week in 5 years’ time. This trend could be understood as an awareness by the graduates of the pressure and workload they are expected to undertake. The reality of GP work, where doctors commonly face long working hours, high levels of stress, and potential burn out,^23^ contributes towards the lack of attractiveness of full-time employment. Part-time employment is often found to be an appropriate response to an increasingly difficult work environment, in order to retain high professional standards and personal wellbeing.^24^

## Strengths and limitations

The surveys conducted at three time points (2014, 2015, and 2017) included a wide range of graduation years (from 2010 to 2016) in order to track how perceptions changed over time. A rich variety of questions allowed a thorough analysis of the areas of interest, such as emigration and employment plans.

Although the response rates were respectable, reaching an overall percentage of 38.2%, a decline over time was noted, and therefore this potentially could produce a response bias. The response rate in 2014 was almost double that in 2015 and 2017. The reasons for this are unclear, but it could be due to the novelty of the survey topic the first time it was undertaken, or because there was a particular negative climate around government policy at that time, which encouraged more to respond.

Since there was no survey undertaken in 2016, a lack of data from that year potentially created discontinuity, which if it were present would contribute to stronger evidence. Furthermore, the surveys were self-reported and contained questions based on recall of past events (such as experiences and reasons), which could have led to recall bias. When investigating the reasons for emigration, the responders were given seven options from which they could choose. However, limiting their answer to one of these options only could be seen as a limitation, especially considering that multiple reasons may have led to their decision to emigrate. GP graduates who were abroad had the same opportunity to respond as the GPs located in Ireland, and hence there is unlikely to be a bias in this regard.

## Comparison with existing literature

The authors believe this is the first study of its kind in Ireland to document the career intentions of recent graduates of specialist GP training. There has been a lack of international literature which investigates intentions of GP graduates in recent years. However, this study complements findings from Australian and UK studies, where GP registrars and newly qualified GPs intended to work part-time hours in the future, especially due to the problems which arise as a result of work–life imbalance.^25,26^

In 1992, 97% of GPs in Ireland were in full-time practice.^27^ By 2015, this had reduced to 84%, and this survey forecasts a fall below 50%, irrespective of sex,^27^ unless the current trend is investigated and intervention occurs which focuses on factors influencing hours worked by GPs in Ireland. Given the predicted rates of retirement of largely full-time GPs^1,18 ^and the proportion of graduates who do not plan to work full time, it is likely that there will be a further supply issue due to insufficient GP replacement.

## Implications for research

Further work is required to investigate the lack of desire among responders for the traditional non-clinical roles of GPs in Ireland. The National Association of General Practitioners believes that the Irish health service does not provide the necessary premises, practice management, administrative staffing, and information technology for a general practice infrastructure.^28^ Traditionally, this has been provided by the GP principal or principals. As a consequence, recent Irish GP graduates wish to work significantly fewer hours, with little desire to run the infrastructure.

It is Canadian state policy to attract medical school graduates into family medicine (presently 40%) through their College of Family Practitioners Canada.^29^ It is well documented how ‘push–pull’ factors influence the emigration of Canadian graduates to the US.^30^ Similar analysis could be applied to the Irish situation. Among recent graduates who had emigrated at the time of the present survey, there is an opportunity to establish what might attract them back to work in Ireland and, furthermore, what might encourage their colleagues working in Ireland to stay.

In conclusion, if current Irish national healthcare policy is to be successfully implemented, a sufficient and sustainable workforce is required, particularly in terms of improving patient care and moving more aspects of care out of hospitals to primary care. Given the numbers of mostly full-time GPs due to retire,^1^ and the apparent insufficient replacement ratios by a large proportion of part-time GPs, it is clear that government policy must change in terms of GP training and their infrastructural procurement policy. The move of young Irish GPs abroad supports evidence that, in order to address GP retention, issues such as number of GPs per capita and working hours are critical factors.^16,17,31^ A policy to retain young GPs, particularly in the first 2 years after graduation, should be considered. However, this is only one part of the equation; further research is needed in the context of retention and returning policies for more mature GPs.
